# Delayed brain metastasis after 17-years from papillary thyroid cancer without local recurrence: case report and literature review

**DOI:** 10.1093/jscr/rjac215

**Published:** 2022-05-31

**Authors:** James Knight, Matthew Kingham, Sally-Ann Price, Istvan Bodi, Jose Pedro Lavrador

**Affiliations:** 1 Department of Neurosurgery, King’s College Hospital, London, SE5 9RS, UK; 2 GKT School of Medical Education, King’s College London, London, SE1 1UL, UK; 3 Department of Neuropathology, King’s College Hospital, London, UK

## Abstract

Papillary thyroid carcinoma (PTC) is the most common malignancy originating from the thyroid, with a good overall prognosis. However, distant metastasis of such lesions is very rare, with the brain being an incredibly uncommon site for secondary spread. The authors report a case of PTC brain metastasis 17-years after successful treatment of the primary malignancy, with no local or locoregional recurrence. Initial diagnostic uncertainty necessitated the involvement of a multidisciplinary team, and eventually the patient underwent image-guided gross surgical resection with intraoperative neuromonitoring (IOMN).

## INTRODUCTION

Papillary thyroid carcinoma (PTC) is the most common malignancy originating from the thyroid [1]. It is of epithelial origin with follicular differentiation. PTC typically presents in middle age, has a 3:1 female predominance and generally carries a good overall prognosis [2]. It can invade local lymphatic systems, with ~10% of patients presenting with synchronous metastatic disease. Epidemiologically, it has a positive correlation with ionising radiation exposure, particularly childhood exposure, and high dietary iodine intake [3]. However, the incidence of distant metastasis (DM) has been found to be as low as 0.1% [4], with the most common sites being lung and bone; the brain being exceptionally rare.

We report a case of PTC brain metastasis 17-years after successful treatment of the primary malignancy with no local or locoregional recurrence. Initial diagnostic uncertainty necessitated considerable multidisciplinary teamwork between oncologists, radiologists, neurosurgeons, head and neck multidisciplinary team (MDT) and lung MDT, which eventually provided an integrated diagnosis and treatment plan.

## CASE PRESENTATION

A 73-year-old lady with a background of papillary thyroid cancer with lung metastases presented to the stroke team with sudden onset left-sided facial weakness. The patient underwent a plain CT head which demonstrated a right frontal lesion that was compressing the right lateral pre-central sulcus. This was consistent with the presentation of left-sided facial weakness. She was noted to have no primary lesion on CT chest, abdomen, and pelvis. Her facial palsy improved with the administration of corticosteroids.

MRI brain with contrast was felt, by the local oncology MDT, to demonstrate a metastatic lesion, with the favoured differential of an undiagnosed primary lung tumour. However, no evidence of a primary lesion was confirmed on FDG CT PET and hence she was discussed at the Network Thyroid cancer MDT. 17-years previously, she had been treated for metastatic papillary thyroid carcinoma with lung metastases, which was refractory to radioiodine therapy. At that time, she underwent a total thyroidectomy and right sided radical neck dissection with post-operative radioiodine remnant ablation and radiotherapy. Post-therapy imaging suggested no iodine avid disease.

**Figure 1:**
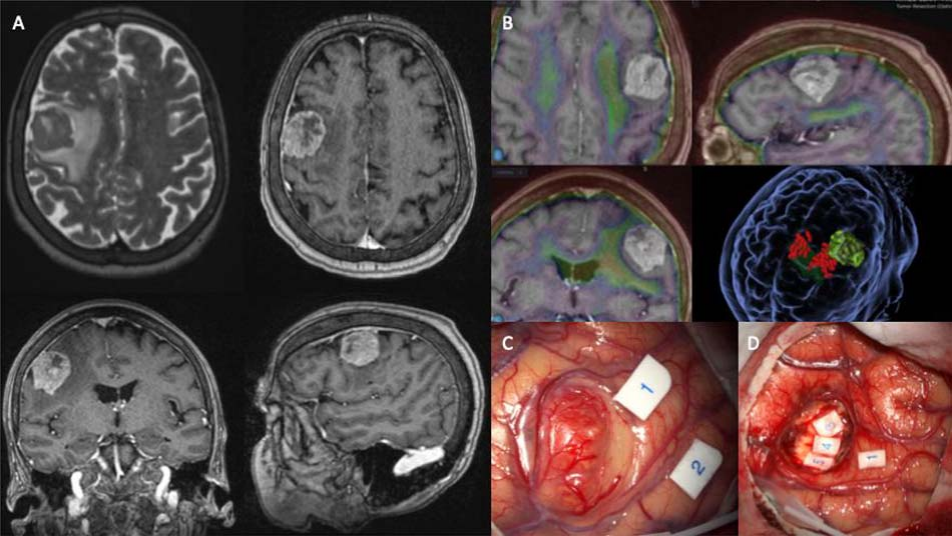
(**A**) shows MRI sequences (top left) T2 axial, (top right) T1 post gadolinium axial, (bottom) T1 post-gadolinium coronal and sagittal. (**B**) 3D preoperative modelling and MRI-PET FDG Fusion. Light is tumour and dark green is cortico-spinal tracts. Red dots are positive motor responses – upper and lower limbs – during preoperative motor mapping with navigated transcranial magnetic stimulation. (**C** and **D**) show intraoperative microscope images of the tumour (**C**) and the post-resection tumour cavity (**D**). Tags 1 and 2 show the motor areas for tongue. At the depth of the tumour cavity (**D**), tag 3 also represents the motor area for the tongue, while tags 4 and 5 represent the *Orbicularis Oris* muscle.

Presently, the patient underwent surgical resection utilising image guidance systems to provide accurate localisation of the tumour and minimise incisions when accessing the lesion. Part of this guidance imaging is shown in Fig. 1B. The surgical team also utilised intraoperative neuro-monitoring (IONM). Motor-evoked potentials (MEP) are shown in Fig. 1C and D. Unfortunately, the patient expired 16-weeks postoperatively.

**Figure 2:**
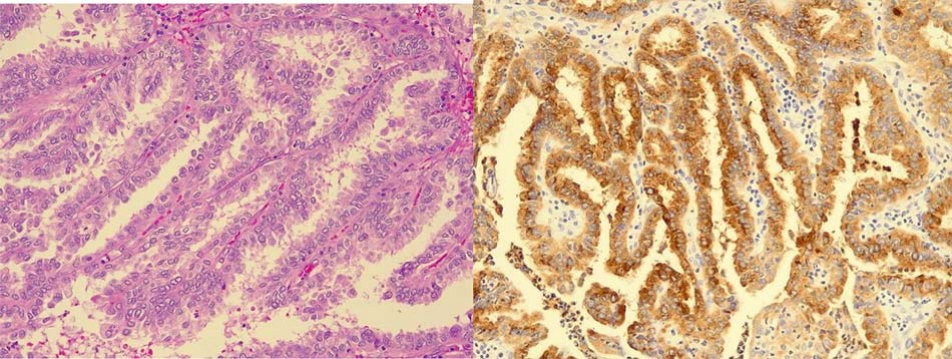
Brain biopsy: (**A**) Metastatic papillary carcinoma, tall cell variant (haematoxylin-eosin). Papillary growth pattern with delicate branching of papillae. The heights of the tumour cells are at least three times their widths and have apical microvacuolation. (**B**) Immunohistochemistry for thyroglobulin is positive.

Brain biopsy showed a metastatic papillary carcinoma with tall columnar cytoplasm and apical microvacuolation (Fig. 2A). Occasional nuclear pseudoinclusions were seen and mitotic figures were rare. Immunohistochemistry demonstrated positivity with CK7, PAX8, Cyclin-D1, thyroglobulin and TTF1 (Fig. 2B). CK20, CDX2, chromogranin, GATA3 and CGCDFP15 were negative. 20 gene targeted next generation sequencing (NGS) 20-gene panel detected BRAF V600E, TERT promoter and PIK3CA mutations. These molecular features and the immunohistochemistry profile were all in keeping with the previous diagnosis of metastatic papillary thyroid carcinoma, favouring a tall cell variant.

## DISCUSSION

PTC is the most common malignancy of thyroid origin, and its incidence is positively correlated with childhood ionizing radiation exposure [1, 3]. However, incidence of DM is rare - a large case-series of n = 8808 patients with PTC found only twelve (0.1%) cases with DM, two (0.01%) of which were brain metastases [4]. Both of those brain metastasis cases also had metastatic lung lesions. What is particularly unique about our reported case is the long, 17-year, latency period between successful treatment of PTC and the presentation of brain metastasis. A literature review found the longest reported duration to be a cerebellar metastasis, presenting 19-years after the primary lesion in an 80-year-old lady who died 7-months later [6]. We also found two other cases with a long latency of 13-years between treatment for the primary tumour and brain metastasis presentation [7, 8].

Further reported cases of PTC brain metastasis include a paper by Tunio *et al.* [6], which presented 7 cases of PTC with cerebellar metastasis ranging from 9-months to 19-years after initial primary diagnosis. The mean duration to metastasis of six cases (19-year case excluded) was 4-years, and five of the seven cases (71%) died due to metastatic disease. Four cases died between 7 and 11-months and one 7-years following the diagnosis of brain metastasis. Guelho *et al.* [9] reported a 43-year-old lady who presented nearly 9-years after primary diagnosis with a frontal metastasis and lived 15-years after diagnosis. Ito *et al.* [1] reviewed 5969 patients with PTC and found 71 (1%) had DM - the majority (n = 60) were in the lung with only one being found in the brain (0.017% of total cohort). This concurred with Ji Jeon *et al.*’s [4] review of 8808 patients with a reported rate of 0.01% of cases presenting with brain metastasis.

Using their 5969-patient sample, Ito *et al.* [1] performed a multivariate analysis of prognostic factors in PTC with DM. Kaplan–Meier and log rank tests were used to produce survival percentages at 5-year and 10-year intervals. Patients aged 55-years or over gave the largest hazard ratio, and overall five-year survival was at least 56%, with 10-year being 40%. Those with massive extra-thyroid extension had the lowest 5-year survival rate and those with DM in organs other than lungs had the lowest 10-year survival.

Extrapolating from these data, our patient had two of the above poor prognostic factors, namely age and DM outside of the lungs. She also had tall cell PTC; a histological variant known to be more aggressive. A study by Dettmer *et al.* showed TERT promoter mutation in tall cell PTC to be highly significant at predicting tumour relapse, while BRAF mutation did not influence patient outcome [5]. Our patient had both mutations on 20 gene targeted NGS. Given more than a 50% probability of 5-year survival, it was felt that our case warranted gross-total resection to give the best chance of a long-term survival. However, due to her risk factors the patient expired 16 weeks post-operatively. Despite this outcome, it was clear from the case that early involvement of MDTs is essential to help inform the management of patients with late recurrences, as the differential diagnosis can be broad.

## CONFLICT OF INTEREST STATEMENT

None declared.

## FUNDING

None.

## ETHICS STATEMENT

Written informed consent for publication of their details was obtained from the patient’s next of kin.
